# Implementation of a self-sampling HPV test for non-responders to cervical cancer screening in Japan: secondary analysis of the ACCESS trial

**DOI:** 10.1038/s41598-022-18800-w

**Published:** 2022-08-25

**Authors:** Misuzu Fujita, Kengo Nagashima, Minobu Shimazu, Misae Suzuki, Ichiro Tauchi, Miwa Sakuma, Setsuko Yamamoto, Hideki Hanaoka, Makio Shozu, Nobuhide Tsuruoka, Tokuzo Kasai, Akira Hata

**Affiliations:** 1 Department of Health Research, Chiba Foundation for Health Promotion and Disease Prevention, 32-14 Shin-Minato, Mihama-ku, Chiba, 261-0002 Japan; 2 Department of Public Health, Chiba University Graduate School of Medicine, Chiba, 260-8670 Japan; 3 Biostatistics Unit, Clinical and Translational Research Center, Keio University Hospital, Shinju-ku, Tokyo, 160-8582 Japan; 4 Research Center for Medical and Health Data Science, The Institute of Statistical Mathematics, Tachikawa, Tokyo 190-8562 Japan; 5 Clinical Research Center, Chiba University Hospital, Chiba, 260-8677 Japan; 6 Department of Health and Welfare, Municipal Health Center, Ichihara, Chiba 290-0050 Japan; 7 Department of Reproductive Medicine, Chiba University Graduate School of Medicine, Chiba, 260-8670 Japan; 8 Yushudai Clinic, Ichihara, Chiba 299-0125 Japan; 9 Center for Preventive Medical Sciences, Chiba University, Chiba, 260-8670 Japan

**Keywords:** Public health, Epidemiology, Randomized controlled trials, Cancer prevention, Cancer screening, Gynaecological cancer

## Abstract

A self-sampling human papillomavirus (HPV) test could improve the morbidity and mortality of cervical cancer in Japan. However, its effectiveness and feasibility have not been demonstrated sufficiently. Hence, we launched a randomized controlled trial, which is ongoing, and report the results of a secondary analysis. To ensure autonomous participation with a minimum selection bias, opt-out consent was obtained from women who met the inclusion criteria, and written consent was obtained from those who underwent a self-sampling test. The number of women who met the inclusion criteria was 20,555; 4283 and 1138 opted out before and after the assignment, respectively. Of the 7340 women in the self-sampling arm, 1372 (18.7%) ordered and 1196 (16.3%) underwent the test. Younger women in their 30 s and 40 s tended to undertake the test more frequently than older women in their 50 s (*P* for trend < 0.001). Invalid HPV test results were rare (1.3%), and neither adverse events nor serious complaints were reported. Despite adopting the opt-out procedure, more women than expected declined to participate, suggesting the need for a waiver of consent or assignment before consent to reduce selection bias. A self-sampling HPV test can be implemented in Japan and would be more accessible to young women, the predominant group affected by cervical cancer.

## Introduction

Cervical cancer is a malignant neoplasm that develops in the cervix and is the fourth most common cancer in women worldwide. Ninety-nine percent of cases are linked to infections caused by the human papillomavirus (HPV) but can be prevented by the adequate implementation of an organized screening program and vaccination against the virus^1,2^. In Japan, efforts to immunize women against HPV had been initiated but reports of adverse events from the vaccination prompted the Ministry of Health, Labor and Welfare (MHLW) to suspend the recommendation to inoculate in June 2013. This drastically lowered the vaccination rate against HPV to almost 0% in 2015^3^. Although the active HPV vaccine recommendations resumed in April 2022^4^, the negative effects of the suspension will remain. Consequently, the strategy to prevent cervical cancer in Japan largely depended on screening. Despite this, the screening rate in Japan remains extremely low compared with that in other developed countries^5^. In 2018, the screening rate was only 16.0% according to the Report on Regional Public Health Services and Health Promotion Services^6^, and it was 43.7% in 2019 based on a self-reported questionnaire as part of the MHLW’s Comprehensive Survey of Living Condition^7^. Given this premise, the incidence of and mortality from cervical cancer in Japan has increased^8,9^, especially among young women^9^.

The current guidelines for cervical cancer screening in Japan recommend only cytology and HPV tests using a sample collected by a doctor. These examinations, however, have ingrained barriers of emotional issues, such as embarrassment and discomfort, and of practical issues, such as lack of time and a laborious collection process^10–13^. Unlike cervical cytology, HPV testing has similar validity between self-collected and physician-collected samples^14,15^ and self-sampling HPV testing can overcome the aforementioned barriers. Randomized trials in various countries have proven that a self-sampling HPV test increased screening uptake^16–21^ and that several countries have already implemented the test as an option for non-responders to screening^22,23^. However, Japan has not yet adopted this method in its current guidelines for cervical cancer screening owing to the lack of scientific evidence for the Japanese population, and limited clinical applications further hinder the adoption of the test.

In line with this, we initiated a large, randomized trial in 2020 called Accelerating Cervical Cancer Elimination by Self-Sampling test (ACCESS) to evaluate the effectiveness of the self-sampling HPV test in screening uptake and precancer detection^24^. As the trial is ongoing, the primary endpoint will be reported in the future. Here, we report the results clarified at present as the secondary analyses of the ACCESS trial which were predetermined in the protocol^24^, discuss issues related to informed consent procedures revealed through the implementation experience, and further evaluate the feasibility of the test in Japan.

## Results

**Figure 1:**
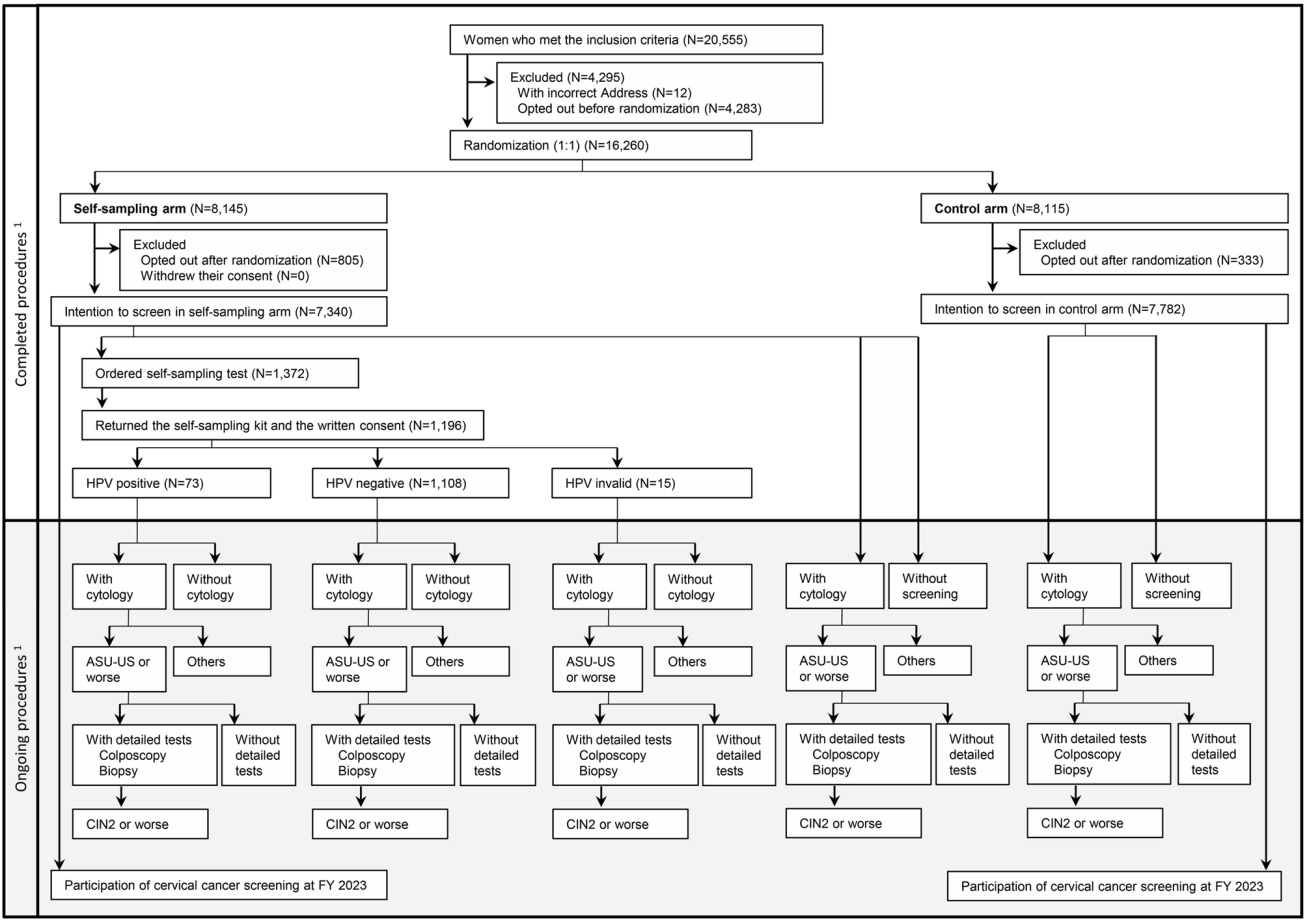
Flowchart of this study. *HPV* human papillomavirus, *ASC-US* atypical squamous cells of undetermined significance, *CIN* cervical intraepithelial neoplasia. ^1^At the time of database lock for this analysis (October 6, 2021).

A flowchart is shown in Fig. 1. The number of women who met the inclusion criteria was 20,555. Of those, women with an incorrect address (N = 12) and those who opted out before assignment (N = 4283) were excluded. The remaining 16,260 women were assigned randomly to the self-sampling arm (N = 8145) and the control arm (N = 8115) at a 1:1 ratio. At the time of database lock for this study, an additional 1,138 women opted out (805 in the self-sampling arm and 333 in the control arm).

The characteristics of the self-sampling arm are shown in Table 1. In the self-sampling arm, 1,372 participants (18.7% with a 95% confidence interval [CI] of 17.8–19.6%) ordered the self-sampling HPV test by July 30, 2021, as shown in Table 2. Of those, 1,213 (88.4% with a 95% CI of 86.6–90.0%) ordered it through the website. Younger participants were more inclined to order the test through the website compared with older participants *(P* < 0.001 in both chi-square test and linear trend test); however, even in the oldest group, the percentage was 80.7% (95% CI 76.5–84.5%). Although we planned to accept sample returns by August 31, 2021, 17 participants submitted their samples after the deadline. All samples were submitted by September 3, 2021. The number of participants who returned the sample was 1,196, the percentage per intention to screen (ITS) was 16.3% (95% CI 15.5–17.2%), and the percentage of those who ordered the test was 87.2% (95% CI 85.3–88.9%). Among the ITS, there were significant inverse associations between age and the proportions of the participants who ordered the test (*P* < 0.001 in both chi-square test and linear trend test) and returned their samples (*P* = 0.001 in chi-square test and *P* < 0.001 in linear trend test). The proportions of the participants in their 30 s and 40 s who returned their samples were similar and were higher than that in participants in their 50 s; therefore, younger participants in their 30 s and 40 s tended to favor self-sampling HPV tests compared to older participants in their 50 s. In addition, a shorter duration of not participating in the screening was associated with a higher frequency of both ordering (*P* < 0.001 in both chi-square test and linear trend test) and returning the sample (*P* < 0.001 in both chi-square test and linear trend test). None of the participants reported any adverse events.

**Table 1:** Participants’ background characteristics in the self-sampling arm.

Number	7340
**Age (years)**	
Mean	44.6
Standard deviation	8.3
Minimum	30
25th percentile	38
Median	46
75th percentile	52
Maximum	58
**Age category (years)**^a^	
30–34	1231 (16.8)
35–39	941 (12.8)
40–44	1401 (19.1)
45–49	1236 (16.8)
50–54	1582 (21.6)
55–59	949 (12.9)
**Duration without screening (years)**^a^	
3–5	773 (10.5)
≥ 6	1467 (20.0)
Without registration	5100 (69.5)

^a^Number (percentage) is shown.

**Table 2:** Percentage of participants who ordered the test, ordered the test through the website, and returned a sample.

	ITS	Ordered self-sampling HPV test kit			Ordered through website			Returned kit				
	N	N	%(95% CI)^a^	*P* value	N	%(95% CI)^b^	*P* value	N	%(95% CI)^a^	*P* value	%(95% CI)^b^	*P* value
Total	7340	1372	18.7(17.8–19.6)		1213	88.4(86.6–90.0)		1196	16.3(15.5–17.2)		87.2(85.3–88.9)	
**Age category (years)**												
30–39	2172	461	21.2(19.5–23.0)	< 0.001^c^	440	95.4(93.1–97.2)	< 0.001^c^	386	17.8(16.2–19.4)	0.001^c^	83.7(80.0–87.0)	0.023^c^
40–49	2637	512	19.4(17.9–21.0)	< 0.001^d^	451	88.1(85.0–90.8)	< 0.001^d^	453	17.2(15.8–18.7)	< 0.001^d^	88.5(85.4–91.1)	0.015^d^
50–59	2531	399	15.8(14.4–17.2)		322	80.7(76.5–84.5)		357	14.1(12.8–15.5)		89.5(86.0–92.3)	
**Duration without screening (years)**												
3–5	773	228	29.5(26.3–32.8)	< 0.001^c^	195	85.5(80.3–89.8)	0.223^c^	203	26.3(23.2–29.5)	< 0.001^c^	89.0(84.2–92.8)	0.647^c^
≥ 6	1467	324	22.1(20.0–24.3)	< 0.001^d^	284	87.7(83.6–91.0)	0.095^d^	282	19.2(17.2–21.3)	< 0.001^d^	87.0(82.9–90.5)	0.353^d^
Without registration	5100	820	16.1(15.1–17.1)		734	89.5(87.2–91.5)		711	13.9(13.0–14.9)		86.7(84.2–89.0)	

*CI* confidence interval, *ITS* intention to screen, *N* number, *HPV* human papillomavirus.

^a^Denominator is the number of ITS.

^b^Denominator is the number of participants who ordered a self-sampling HPV test.

^c^Pearson’s chi-square test.

^d^Linear trend test.

Of 1,196 participants who underwent the test, 73 (6.1% with a 95% CI of 4.8–7.6%) had HPV-positive results as shown in Table 3. Younger age was associated with a higher percentage of HPV positivity (*P* = 0.034 in chi-square test and *P* = 0.016 in linear trend test). Of the participants who underwent the test, 15 participants (1.3% with a 95% CI of 0.7–2.1%) had invalid results. All of them showed the intention to retake the test, and 11 participants actually returned their samples for the re-test. Results from the re-test yielded seven valid HPV-negative results, one positive result, and three invalid results again. One of the three participants with invalid results underwent a third examination (the remaining two were not willing to undergo further testing by self-sampling) and obtained valid negative results for HPV.

**Table 3:** Self-sampling human papillomavirus test results.

	All^a^	HPV test result						Details of positive results		
		Invalid			Positive			Type 16	Type 18	Others^d^
	N	N	%(95% CI)	*P* value	N	%(95% CI)	*P* value	N	N	N
Total	1196	15	1.3 (0.7–2.1)		73	6.1 (4.8–7.6)		13	5	58
**Age category (years)**										
30–39	386	5	1.2 (0.4–3.0)	0.963^b^	29	7.5 (5.1–10.6)	0.034^b^	4	3	23
40–49	453	6	1.3 (0.5–2.9)	0.828^c^	32	7.1 (4.9–9.8)	0.016^c^	7	1	26
50–59	357	4	1.1 (0.3–2.8)		12	3.4 (1.7–5.8)		2	1	9
**Duration without screening (years)**										
3–5	203	3	1.5 (0.3–4.3)	0.573^b^	14	6.9 (3.8–11.3)	0.703^b^	3	0	12
≥ 6	282	5	1.8 (0.6–4.1)	0.554^c^	19	6.7 (4.1–10.3)	0.499^c^	5	0	14
Without registration	711	7	1.0 (0.4–2.0)		40	5.6 (4.0–7.6)		5	5	32

*CI* confidence interval, *HPV* human papillomavirus, *N* number.

^a^Participants who underwent the self-sampling test.

^b^Pearson’s chi-square test.

^c^Linear trend test.

^d^Pooled high-risk HPV types including types of 31, 33, 35, 39, 45, 51, 52, 56, 58, 59, 66, and 68.

The time-to-event results are shown in Table 4 and Supplementary Fig. S1. It took only 9 days for half of the participants to order the test and 89 days for 90% of them, whereas it took a relatively long time for participants to return the samples after receiving the kit; it took 35 days for half of them and 133 days for 90% of them. The period between collecting a sample and returning it was quite short. Of 1,194 participants who returned a sample and declared the sample collection date, 9 (0.75%) participants were outside of the recommendation, meaning they submitted their sample past the 1-week recommendation according to the instruction manual created by a kit vendor in Japan (Harada Corporation, Osaka, Japan)^25^. In particular, one participant took 27 days, and the test result was invalid. In contrast, the remaining 14 participants with invalid test results returned their samples within 2–4 days after sample collection. We did not receive any complaints from the participants in terms of the procedures or time schedule.

**Table 4:** Time to each event.

Depend on	Time to event (95% CI)							
	To order the test^a^	To send a kit^b^	To return a sample since sending the kit^c^	To return a sample since collecting it^d^	To order the test to the laboratory^e^	Time for the laboratory to report the results^f^	To send the results to participants ^g^	Total time ^h^
	Participants	Researchers	Participants	Participants	Researchers	Laboratory	Researchers	―
Number	1372^i^	1372^i^	1196^j^	1194^k^	1196^j^	1196^j^	1,196 ^j^	1,196 ^j^
Minimum	1	2	4	1	1	6	1	29
10th percentile	2 (2–2)	4 (4–4)	7 (6–7)	2 (2–2)	1 (1–1)	10 (10–10)	2(2–2)	36(36–36)
20th percentile	3 (2–3)	5 (5–5)	11 (10–11)	2 (2–2)	1 (1–1)	11 (11–11)	2(2–2)	43(43–43)
30th percentile	4 (3–4)	6 (5–6)	17 (14–18)	2 (2–3)	1 (1–1)	11 (11–11)	2(2–2)	65(49–65)
40th percentile	5 (5–6)	6 (6–6)	25 (21–27)	3 (3–3)	1 (1–1)	12 (12–12)	2(2–2)	72(65–79)
50th percentile	9 (8–10)	7 (7–7)	35 (33–39)	3 (3–3)	1 (1–1)	12 (12–13)	3(3–3)	93(85–93)
60th percentile	15 (13–18)	7 (7–7)	49 (46–53)	3 (3–3)	1 (1–1)	13 (13–13)	3(3–3)	114(106–120)
70th percentile	27 (23–32)	7 (7–8)	62 (60–68)	4 (4–4)	1 (1–1)	13 (13–14)	3(3–3)	141(133–155)
80th percentile	53 (47–58)	8 (8–9)	94 (82–102)	4 (4–4)	1 (1–2)	14 (14–14)	3(3–3)	161(161–169)
90th percentile	89 (83–92)	9 (9–10)	133 (129–137)	5 (5–5)	3 (3–3)	15 (15–15)	4(4–4)	183(176–183)
Maximum	112	17	168	27	5	22	4	198

Time to event was expressed in days.

*CI* confidence interval.

^a^Between the time that the second invitation letter was sent to the participants and the time that the self-sampling human papillomavirus (HPV) test kit was ordered by the participants.

^b^Between the time that the self-sampling HPV test was ordered and the time that the kit was sent to the participants.

^c^Between the time the kit was sent to the participants and the time a sample was returned.

^d^Between the time that a sample was collected by the participants and the time the sample was returned.

^e^Between the time that a sample was returned by the participants and the HPV test was ordered to the laboratory.

^f^Between the time that the HPV test was ordered to the laboratory and the results were reported by the laboratory.

^g^Between the time that the results were reported by the laboratory and the results were sent to the participants.

^h^Between the time that the second invitation letter was sent to the participants and the time that the HPV test results were sent to the participants.

^i^The participants to be analyzed were those who ordered the self-sampling HPV test.

^j^The participants to be analyzed were those who returned both the sample and their written consent.

^k^The participants to be analyzed are those who returned both the sample and their written consent and reported the data collected from a sample.

## Discussion

Through the ACCESS trial, we identified issues related to the informed consent procedure in evaluating population-based interventions such as screening. Overall, we obtained useful results to implement the self-sampling HPV test in practice.

In general, randomized controlled trials require written consent from participants before assignment. However, this kind of conventional consent may not be suitable for evaluating population-based interventions such as screening, where it is important to estimate the effects on the whole population^26^. In almost all of the previous trials on the effectiveness of the self-sampling HPV test, random assignment took place without consent, and only participants who underwent the screening accordant with the allocated arm gave written consent^12,13,19,20,27–29^, in which Zelen’s design was included^27^. In other previous trials, although few, the requirement for informed consent was waived^16,21,30^. In the current study, we adopted a more participant-centered method to obtain informed consent to follow the ethical guidelines for medical and health research involving human subjects in Japan and the opinions of the Committee for Personal Information Protection of Ichihara City. In detail, all participants provided opt-out consent before the assignment, and those who underwent self-sampling HPV testing provided written informed consent. A similar method was adopted in a previous study conducted in the Netherlands^31^. The percentage of women who opted out before assignment in our trial (20.8%) was higher than that in the previous study (15.1%)^31^. Since there is no nationwide database for cervical cancer screening in Japan, unlike in the Netherlands, true non-responders to screening are obscure. Therefore, some of the women who met the inclusion criteria might have had the opportunity to receive cervical cancer screening provided by their affiliations, such as workplaces, other than the city. In such cases, women might naturally decline to participate in this trial. Other reasons for the high number of participants who opted out, such as distrust of the research institution and anxiety about personal information leakage, might be plausible. A substantial number of participants opting out before random assignment can cause selection bias. If women who are interested in cervical cancer screening participate more frequently in trials, the screening uptake might be overestimated. In addition, participants opted out throughout the study period, i.e., an additional 805 in the self-sampling arm and 333 in the control arm opted out after assignment at the time of the database lock. The number of women who opted out was higher in the self-sampling arm than in the control arm, and one of the factors might be the second invitation letter sent to the self-sampling arm. Consequently, confounding factors might have been induced. Considering these results, we suggest that random assignment before obtaining consent as per Zelen’s design and waiver of consent should be accepted in Japan, as previous studies in other countries have already adopted this design where the risk to participants was considered to be minimal compared with the scientific benefit.

No adverse events were reported in this trial, which is consistent with a previous trial with a small sample size in Japan^32^. Additionally, the occurrence of invalid results was rare (1.3%), which was similar to most of the previous studies in various countries (0.27–1.36%)^13,19–21,33,34^ except for one (11.8%)^29^. We confirmed the safety of the self-sampling device and the practical utility of the device and the HPV test in Japan. According to previous questionnaire surveys, about 10–20% of women were not confident in their self-sampling procedure^10,31,35–37^. Providing these results to women could be useful to alleviate their anxiety when they collect their samples by themselves, especially for the first time.

Of the participants in the self-sampling arm, 18.7% ordered the test and 16.3% returned their samples in this trial, which were similar to previous studies where the ordering rates were 12.5–31.7% and returning rates were 8.2–20.4%^17,18,29,38–40^. Considering that the subjects of this study were non-responders to cervical cancer screening, these rates are meaningful, but not high in value. When conducting a self-sampling HPV test, kits, specimens, and test results are generally sent by mail. In Japan, mailboxes are placed everywhere, so accessibility should not be an issue. Additionally, according to the previous questionnaire surveys, self-sampling HPV tests were recognized to be convenient and easy for women^10,28,31,37,41,42^. Therefore, the complexity of the procedures may not be a barrier to conducting a self-sampling HPV test. Although we cannot clarify the reasons for not undergoing the test, other factors, such as anxiety about the test accuracy, a fear of inexperience in self-testing, concerns about privacy protection, and trust in the institution, might be related. The fact that a relatively large population did not respond to self-sampling HPV tests suggests the need for multipronged efforts to improve screening uptake.

Additionally, we found that most participants ordered the test through the website, not telephonically, despite providing a toll-free telephone number for this trial to avoid financial burden on the participants. The lack of a time restriction might have enhanced orders through the website; in contrast, orders by phone were restricted to a given period from 9:00 a.m. to 5:00 p.m. in this trial. Furthermore, the ownership rate of mobile devices in Japan, such as smartphones, personal computers, and tablets, was 96.1% in 2019^43^. Thus, orders through the website were thought to be familiar to most participants. A previous study conducted in Denmark reported similar results. The study provided four methods to order the test: through the website, by phone, by mail, and by e-mail; ordering through the website was the most preferred method followed by mail^18^. In another previous study, mail was the most preferred method, followed by ordering through the website^38^. Since we did not provide mail as an option to order the test, we do not know its effect in this trial. This method might be useful for improving the ordering rate, especially among women who do not own mobile devices.

There are two well-known strategies for the self-sampling HPV test: the on-demand strategy, in which the kits are sent to the subjects on demand, and the direct mail strategy, in which the kits are sent to all subjects. Our trial adopted the on-demand strategy. Among the participants who ordered the test, 87.2% returned a sample, which seems to be higher than those in previous studies, which ranged from 60.4 to 79.0%^17,18,29,38–40^. The on-demand strategy has the disadvantage of a lower response rate compared with the direct mail strategy^17,18,29,40^, but it has the advantage of reducing device waste and costs, such as for shipping and device purchase. Considering the limited budgets of the municipalities of Japan, the direct mail strategy might not be viable.

The association between age and the proportion of participants who underwent self-sampling HPV testing was inconsistent. Some studies reported a positive association^29,38^, whereas others reported a convex curve association^31,37^ or no association^18–20,39,44^. This trial revealed a significant inverse association between age and the proportion of participants. Cervical cancer predominantly occurs in women in their 30 s and 40 s^8^. Additionally, in Japan, the HPV vaccine recommendation had been suspended from June 2013 to March 2022^3,4^, and cervical cancer morbidity and mortality have increased recently^8,9^, especially in young women^9^. Therefore, a measure to improve screening uptake is required, especially for young women. A self-sampling HPV test could be an optional measure for this purpose.

In terms of the time to events analysis, about 3 months would be sufficient to order the test, and 4 to 5 months would be required to return a sample after sending the kit. Most participants returned the kit immediately after sample collection since we asked them to return the kit within 24 h after sample collection. However, one woman took up to 27 days to return the kit, and her test results were invalid. Although one of the reasons for invalid results might be a long period from sample collection to testing, not all of the reasons are apparent. Deviations from other manufacturer recommendations for the kit, including prohibition of storage in high temperature and humidity, touching the collection part, and vaginal cleaning before collection, might be involved. No participant complained about the procedures and time schedule, presumably because we could provide services within a relatively short time.

There were some limitations. First, several women who met the inclusion criteria refused to participate in this trial. Therefore, the proportion of participants who underwent the test might have been overestimated. Second, the generalizability of the results is limited. The participants in this trial were restricted to women living in Ichihara City located about 35–45 km southeast of Tokyo. The area of the city is relatively large (368.17 km^2^) and includes factory, commercial, and rural areas. The population of the city was 274,656 in 2015. The percentage of residents over the age of 65 years was 25.8%, which is similar to that in Japan overall (26.6%). The cervical cancer screening rate was also similar to that of the national average in 2018 (14.8% vs. 16.0%) according to a report on regional public health services and health promotion services^45^. Considering these characteristics of the city, it is expected that the residents in the city would not be greatly different from the population of Japan as a whole. Fourth, there is no registration system for cervical cancer screening in Japan. Therefore, we were unable to extract the true number of unscreened women. Fifth, although the subjects of this trial were the usual target population for cervical cancer screening, the response in women may differ between when the screening is performed as a study and as a practice. However, it is not recommended to perform a screening without evidence as a practice. Therefore, at this time, it is important to establish the evidence as a study in Japan.

In conclusion, despite employing opt-out consent, a substantial number of women refused to participate in this trial, suggesting the necessity of a waiver of consent or assignment before consent, such as in Zelen’s design in order to reduce selection bias. A self-sampling test is an accessible tool for young women in their 30 s and 40 s, who are the predominant group affected by cervical cancer. Ordering kits through the website was preferred by all age groups compared with phone calls. The findings of the ACCESS trial, i.e., no adverse events, rare invalid HPV test results, and no complaints from participants regarding procedures and time schedule, support the implementation of self-sampling HPV tests in Japan as a practice.

## Methods

We report the results of the secondary analyses of the ACCESS trial, an ongoing randomized trial in Japan. This study was registered in the Japan Registry of Clinical Trials (JRCT, 1030200276), and the protocol details have been published previously^24^.

### Participants

The inclusion criteria in the ACCESS trial were as follows: (1) women who resided in Ichihara City on December 22, 2020; (2) women aged between 30 and 59 years as of April 1, 2021; (3) target population for cervical cancer screening by Ichihara City in 2021; and (4) women who had not undergone routine cervical cancer screening provided by Ichihara City for ≥ 3 years.

On December 22, 2020, women who met all of the inclusion criteria were extracted from the database of Ichihara City Hall. We sent them a pre-invitation letter on February 1, 2021. The content of the letter specified that they could refuse to participate in the trial and the subsequent procedure. On February 22, 2021, women with a returned pre-invitation letter due to an incorrect address or those who opted out were excluded from the study. The remaining subjects were randomly assigned to the self-sampling arm and the control arm at a 1:1 ratio using computer-generated random numbers. The subjects who were assigned to the self-sampling arm could undergo regular screening (cytology test) or screening with a self-sampling HPV test, and those who were assigned to the control arm could undergo regular screening. Completed and ongoing procedures are shown in Fig. 1. Although the questionnaire survey was also sent to participants who underwent a self-sampling HPV test, the results will be reported elsewhere. The subjects of this study were mainly the participants assigned to the self-sampling arm.

### Completed procedures in the self-sampling arm at the time of database lock for this analysis

The second invitation letter, which was disclosed in the protocol^24^, was sent on March 10, 2021 to the participants assigned to the self-sampling arm. The letter indicated that they could receive either the regular screening (cytology test) or screening with a self-sampling HPV test, along with the instructions on how to order the test. Additionally, the letter included educational information, such as the cause of cervical cancer, preventive methods, currently recommended cervical cancer screening, the low screening rate in Japan, and the validity of self-sampling HPV tests. The test order was placed either by phone or through the website by June 30, 2021. At the time of ordering, in both methods, submission of the personal identity number assigned for this trial, name, and telephone number were required. When ordering through the website, we also asked participants to check boxes to make sure that the following situations did not apply: (1) pregnancy, (2) previous hysterectomy, or (3) no sexual experience. For the participants who ordered the test, a self-sampling kit (Evalyn Brush, Rovers Medical Devices, Oss, the Netherlands), an instruction manual created by a kit vendor in Japan (Harada Corporation, Osaka, Japan) including instructions on how to take a sample^25^, a booklet including how to send a sample, an informed consent form, a questionnaire, and a prepaid envelope for returning the sample were sent by mail. According to an instruction manual, it was recommended that a sample was submitted within 1-week, a sample was not stored in high temperature and humidity, the collection part was not touched, and vaginal cleaning was not performed before collection. The participants were asked to take a sample by themselves and return the sample, a filled consent form, and a filled questionnaire to the Chiba Foundation for Health Promotion and Disease Prevention by mail by August 31, 2021. Participants who ordered the test but had not returned the sample were sent a reminder letter on July 21, 2021. The samples were sent to the laboratory of an outsourced company (LSI Medience Corporation, Tokyo, Japan) for HPV testing with a cobas 8800 system (Roche Diagnostics, Rotkreuz, Switzerland). When the HPV test result was invalid, the laboratory performed a retest up to two times. The HPV test results that were positive, negative, or invalid for each type of HPV (type 16, 18, and 12 other pooled high-risk HPV types including types 31, 33, 35, 39, 45, 51, 52, 56, 58, 59, 66, and 68) were reported to the Chiba Foundation for Health Promotion and Disease Prevention by the laboratory. The participants were also informed of the results by mail through an outsourced company (Accelight Inc., Tokyo, Japan). In the result notice, we recommended that all participants who underwent the self-sampling HPV test undergo regular cervical cancer screening (cytology test) provided by the city since the self-sampling HPV test was not recommended in the guidelines in Japan. For participants with invalid results, we offered additional opportunities for retesting.

### Ongoing procedures in both arms

Regular cervical cancer screening (cytology test) provided by the city was performed until March 31, 2022. If the result of the cytology test was atypical squamous cells of undetermined significance or worse, detailed tests such as colposcopy and biopsy were performed by the medical institutions following an invitation from the city. The results of regular cervical cancer screening (cytology) and detailed tests are administered by the city. The results of cytology tests performed between May 1, 2021 and March 31, 2022, will be provided by March 31, 2023 by the city for this trial. The results of detailed tests performed between May 1, 2021 and March 31, 2023 will be provided by March 31, 2024. Additionally, to determine the screening uptake in 2023, data on whether each participant underwent cytology tests between May 1, 2023 and March 31, 2024 will be provided by March 31, 2025.

### Statistical analysis

We locked the database for this secondary analysis of the ACCESS trial on October 6, 2021. The endpoints and definitions are shown in the Supplementary Method S1. For endpoints except for time-to-event, the frequency and percentage with 95% CIs were calculated. A subgroup analysis of age category and duration without screening was also performed. Age categories were 30–39 years, 40–49 years, and 50–59 years, and the duration without screening was 3–5 years, ≥ 6 years, and without registration. For the subgroup analysis, the frequency and percentage with 95% CIs were calculated for each category, and Pearson’s chi-square test and linear trend test by logistic regression analysis were performed. For time-to-event, summary statistics for survival time (minimum and maximum time and values per 10^th^ percentile with 95% CIs) were calculated, and Kaplan–Meier plots were drawn. All statistical analyses were performed using STATA software (version 15.0; Stata Corp LLC, College Station, TX). Statistical significance was set at *P* < 0.05.

### Ethics declarations

To ensure autonomous participation and reduce selection bias as much as possible, opt-out consent was obtained from all participants. In detail, we sent a pre-invitation letter to all women who met the inclusion criteria, with the option to opt out. Additionally, we received written consent from participants who underwent self-sampling HPV testing. The procedure complies with the Ethical Guidelines for Medical and Health Research Involving Human Subjects in Japan. This trial was approved by the Research Ethics Committees of the Chiba Foundation for Health Promotion and Disease Prevention (approval number R2-2), Graduate School of Medicine, Chiba University (approval number 3979) and the Institute of Statistical Mathematics (approval number ISM20-001). Since Ichihara City does not have an ethics committee, the Committee of the Chiba Foundation for Health Promotion and Disease Prevention reviewed the protocol instead of Ichihara City (approval number R2-7). The Committee for Personal Information Protection of Ichihara City reviewed the plan of this trial on November 12, 2020 and authorized data provision on December 15, 2020. This trial has been performed in accordance with the principles of the Declaration of Helsinki and Ethical Guidelines for Medical and Health Research Involving Human Subjects.

## Supplementary Information

Supplementary Information 1.

Supplementary Information 2.

## Data Availability

The datasets analyzed during the current study are not publicly available in line with the opinions of the Committee for Personal Information Protection of Ichihara City because the consent for sharing the data of the participants was not obtained, but are available from the corresponding author on reasonable request.

## References

[1] Hall MT (2019). The projected timeframe until cervical cancer elimination in Australia: A modelling study. Lancet Public Health.

[2] Simms KT (2019). Impact of scaled up human papillomavirus vaccination and cervical screening and the potential for global elimination of cervical cancer in 181 countries, 2020–99: A modelling study. Lancet Oncol..

[3] Hanley SJ, Yoshioka E, Ito Y, Kishi R (2015). HPV vaccination crisis in Japan. Lancet.

[4] Normile D (2022). Japan reboots HPV vaccination drive after 9-year gap. Science.

[5] OECD. OECD Health Statistics. http://www.oecd.org/els/health-systems/health-data.htm (2019).

[6] Ministry of Health, Labour and Welfare. The report on regional public health services and health promotion services. https://www.mhlw.go.jp/toukei/saikin/hw/c-hoken/18/dl/kekka2.pdf (2018).

[7] Cancer Information Service, National Cancer Center, Japan. Cancer registry and statistics. https://ganjoho.jp/reg_stat/statistics/dl_screening/index.html#a16.

[8] Foundation for Promotion of Cancer Research. Cancer Statitics in Japan. https://ganjoho.jp/data/reg_stat/statistics/brochure/2021/cancer_statistics_2021.pdf (2020).

[9] Yagi A (2019). Epidemiologic and clinical analysis of cervical cancer using data from the population-based Osaka Cancer Registry. Cancer Res..

[10] Sultana F (2015). Women's experience with home-based self-sampling for human papillomavirus testing. BMC Cancer.

[11] Bosgraaf RP (2014). Reasons for non-attendance to cervical screening and preferences for HPV self-sampling in Dutch women. Prev. Med..

[12] Szarewski A (2011). HPV self-sampling as an alternative strategy in non-attenders for cervical screening: A randomised controlled trial. Br. J. Cancer.

[13] Darlin L (2013). Comparison of use of vaginal HPV self-sampling and offering flexible appointments as strategies to reach long-term non-attending women in organized cervical screening. J. Clin. Virol..

[14] Polman NJ (2019). Performance of human papillomavirus testing on self-collected versus clinician-collected samples for the detection of cervical intraepithelial neoplasia of grade 2 or worse: A randomised, paired screen-positive, non-inferiority trial. Lancet Oncol..

[15] Onuma T, Kurokawa T, Shinagawa A, Chino Y, Yoshida Y (2020). Evaluation of the concordance in HPV type between self- and physician-collected samples using a brush-based device and a PCR-based HPV DNA test in Japanese referred patients with abnormal cytology or HPV infection. Int. J. Clin. Oncol..

[16] Winer RL (2019). Effect of mailed human papillomavirus test kits vs usual care reminders on cervical cancer screening uptake, precancer detection, and treatment: A randomized clinical trial. JAMA Netw. Open.

[17] Elfstrom KM (2019). Increasing participation in cervical screening by targeting long-term nonattenders: Randomized health services study. Int. J. Cancer.

[18] Tranberg M (2018). Preventing cervical cancer using HPV self-sampling: Direct mailing of test-kits increases screening participation more than timely opt-in procedures: A randomized controlled trial. BMC Cancer.

[19] Gok M (2010). HPV testing on self collected cervicovaginal lavage specimens as screening method for women who do not attend cervical screening: Cohort study. BMJ.

[20] Gok M (2012). Experience with high-risk human papillomavirus testing on vaginal brush-based self-samples of non-attendees of the cervical screening program. Int. J. Cancer.

[21] Haguenoer K (2014). Vaginal self-sampling is a cost-effective way to increase participation in a cervical cancer screening programme: A randomised trial. Br. J. Cancer.

[22] Polman NJ, Snijders PJF, Kenter GG, Berkhof J, Meijer CJLM (2019). HPV-based cervical screening: Rationale, expectations and future perspectives of the new Dutch screening programme. Prev. Med..

[23] Pedersen K (2018). An overview of cervical cancer epidemiology and prevention in Scandinavia. Acta Obstet. Gynecol. Scand..

[24] Fujita M (2022). Study protocol of the ACCESS trial: A randomised trial to evaluate the effectiveness of human papillomavirus testing by self-sampling in cervical cancer screening uptake and precancer detection. BMJ Open.

[25] Harada Corporation. Instruction manual for Evalyn Brush. https://medical.haradacorp.co.jp/wp-content/uploads/2021/12/Evalyn%E3%83%96%E3%83%A9%E3%82%B7_%E5%8F%96%E6%89%B1%E8%AA%AC%E6%98%8E%E6%9B%B8_%E7%AC%AC5%E7%89%88.pdf.

[26] Torgerson DJ, Roland M (1998). What is Zelen's design?. BMJ.

[27] Cadman L (2015). A randomized controlled trial in non-responders from Newcastle upon Tyne invited to return a self-sample for Human Papillomavirus testing versus repeat invitation for cervical screening. J. Med. Screen..

[28] Giorgi Rossi P (2011). The effect of self-sampled HPV testing on participation to cervical cancer screening in Italy: A randomised controlled trial (ISRCTN96071600). Br. J. Cancer.

[29] Kellen E (2018). A randomized, controlled trial of two strategies of offering the home-based HPV self-sampling test to non-participants in the Flemish cervical cancer screening program. Int. J. Cancer.

[30] Sultana F (2016). Home-based HPV self-sampling improves participation by never-screened and under-screened women: Results from a large randomized trial (iPap) in Australia. Int. J. Cancer.

[31] Bosgraaf RP (2015). Comparative performance of novel self-sampling methods in detecting high-risk human papillomavirus in 30,130 women not attending cervical screening. Int. J. Cancer.

[32] Yamasaki M, Abe S, Miura K, Masuzaki H (2018). The effect of self-sampled HPV testing on participation in cervical cancer screening on a remote island. Acta Med. Nagasaki.

[33] Bais AG (2007). Human papillomavirus testing on self-sampled cervicovaginal brushes: An effective alternative to protect nonresponders in cervical screening programs. Int. J. Cancer.

[34] Carrasquillo O (2018). HPV self-sampling for cervical cancer screening among ethnic minority women in South Florida: A randomized trial. J. Gen. Intern. Med..

[35] Polman NJ (2019). Experience with HPV self-sampling and clinician-based sampling in women attending routine cervical screening in the Netherlands. Prev. Med..

[36] Virtanen A, Nieminen P, Niironen M, Luostarinen T, Anttila A (2014). Self-sampling experiences among non-attendees to cervical screening. Gynecol. Oncol..

[37] Karjalainen L, Anttila A, Nieminen P, Luostarinen T, Virtanen A (2016). Self-sampling in cervical cancer screening: Comparison of a brush-based and a lavage-based cervicovaginal self-sampling device. BMC Cancer.

[38] Lam JU (2017). Human papillomavirus self-sampling for screening nonattenders: Opt-in pilot implementation with electronic communication platforms. Int. J. Cancer.

[39] Broberg G (2014). Increasing participation in cervical cancer screening: Offering a HPV self-test to long-term non-attendees as part of RACOMIP, a Swedish randomized controlled trial. Int. J. Cancer.

[40] Ivanus U (2018). Randomised trial of HPV self-sampling among non-attenders in the Slovenian cervical screening programme ZORA: Comparing three different screening approaches. Radiol. Oncol..

[41] Wong EL, Cheung AW, Wong AY, Chan PK (2020). Acceptability and feasibility of HPV self-sampling as an alternative primary cervical cancer screening in under-screened population groups: A cross-sectional study. Int. J. Environ. Res. Public Health.

[42] Hanley SJ (2016). HPV self-sampling in Japanese women: A feasibility study in a population with limited experience of tampon use. J. Med. Screen..

[43] Ministry of Internal Affairs and Communications, Japan. White paper 2020. https://www.soumu.go.jp/johotsusintokei/whitepaper/eng/WP2020/2020-index.html (2020).

[44] Virtanen A, Anttila A, Luostarinen T, Malila N, Nieminen P (2015). Improving cervical cancer screening attendance in Finland. Int. J. Cancer.

[45] Ministry of Health, Labour and Welfare Japan. Statistics of Japan. https://www.e-stat.go.jp/stat-search/files?page=1&toukei=00450025&tstat=000001030884 (2018).

