# Supplementary material for: Implementation of a self-sampling HPV test for non-responders to cervical cancer screening in Japan: secondary analysis of the ACCESS trial
Source: Sci Rep. 2022 Aug 25;12:14531. doi: 10.1038/s41598-022-18800-w (PMC9411156; doi:10.1038/s41598-022-18800-w)
Supplement: Supplementary file 1 — Supplementary Information 1. [file 41598_2022_18800_MOESM1_ESM.pdf]

Supplementary Figure

Title: Implementation of a self-sampling HPV test for non-responders to cervical cancer screening in Japan: secondary analysis of the ACCESS trial

Authors: Misuzu Fujita[1,2], Kengo Nagashima[3,4], Minobu Shimazu[5], Misae Suzuki[6], Ichiro Tauchi[6], Miwa Sakuma[6], Setsuko Yamamoto[6], Hideki Hanaoka[5], Makio Shozu[7], Nobuhide Tsuruoka[8], Tokuzo Kasai[1], Akira Hata[1,9]

Affiliations: [1]Department of Health Research, Chiba Foundation for Health Promotion and Disease Prevention, Chiba 261-0002, Japan; [2]Department of Public Health, Chiba University Graduate School of Medicine, Chiba 260-8670, Japan; [3]Biostatistics Unit, Clinical and Translational Research Center, Keio University Hospital, Shinju-ku, Tokyo 160-8582, Japan; [4]Research Center for Medical and Health Data Science, The Institute of Statistical Mathematics, Tachikawa, Tokyo 190-8562, Japan; [5]Clinical Research Center, Chiba University Hospital, Chiba 260-8677, Japan; [6]Municipal Health Center, Department of Health and Welfare, Ichihara City, Ichihara, Chiba 290-0050, Japan; [7]Departments of Reproductive Medicine, Chiba University Graduate School of Medicine, Chiba 260-0856, Japan; [8]Yushudai Clinic, Ichihara, Chiba 299-0125, Japan; [9]Center for Preventive Medical Sciences, Chiba University, Chiba 260-0826, Japan

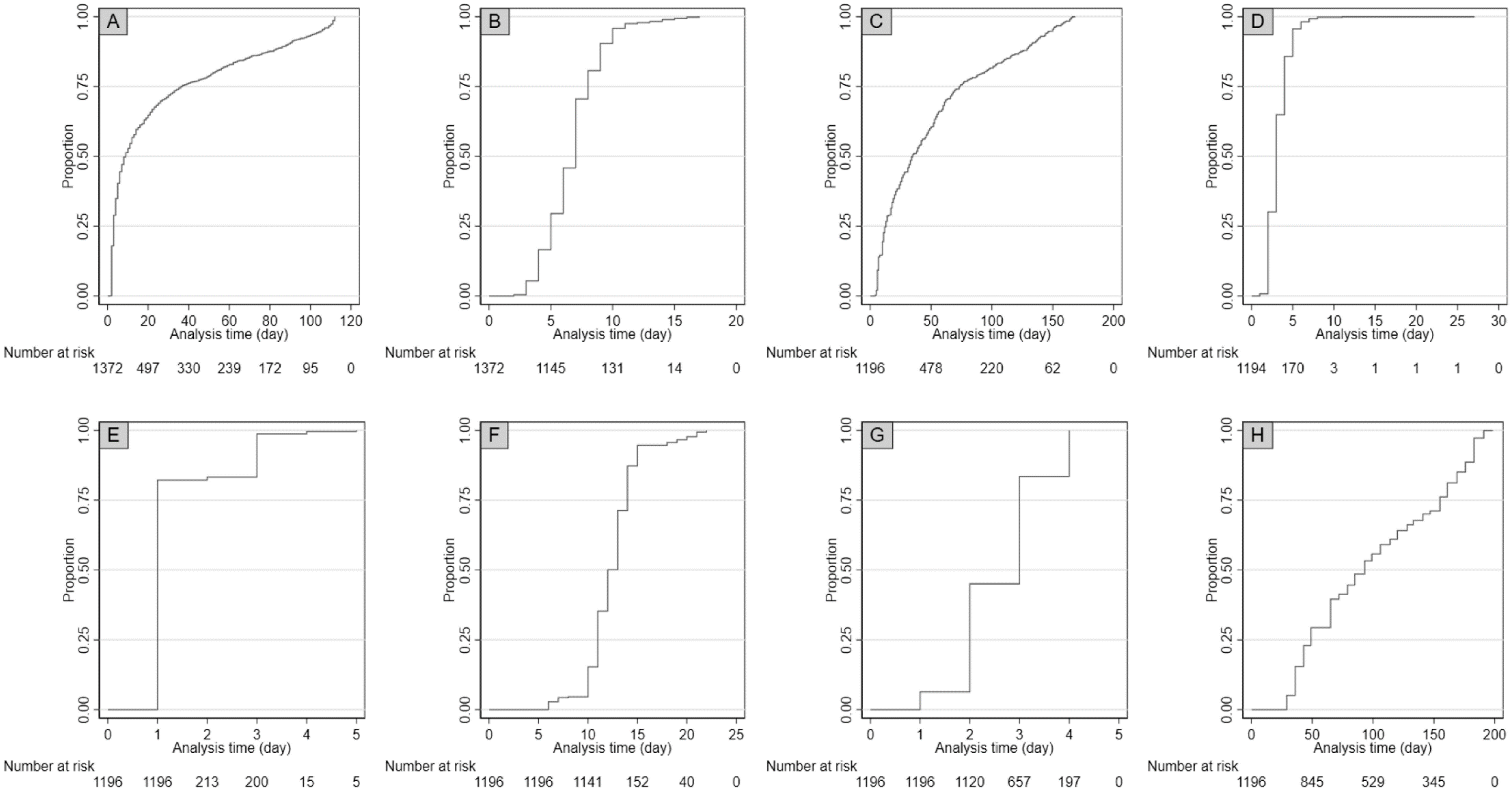

Supplementary Fig. S1

Kaplan-Meier plots for the time to each event

A: Between the time that the second invitation letter was sent to the participants and the time that the self-sampling human papillomavirus (HPV) test was ordered by the

participants.
B: Between the time that the self-sampling HPV test was ordered and the time that the kit was sent to the participants.
C: Between the time that the kit was sent to the participants and the time a sample was returned.
D: Between the time that a sample was collected by the participants and the time a sample was returned.
E: Between the time that a sample was returned by the participants and the HPV test was ordered to the laboratory.
F: Between the time that the HPV test ordered to the laboratory and the results were reported by the laboratory.
G: Between the time that the results were reported by the laboratory and the results were sent to the participants.
H: Between the time that the second invitation letter was sent to the participants and the time that the HPV test results were sent to the participants.
