# Supplementary material for: Implementation of a self-sampling HPV test for non-responders to cervical cancer screening in Japan: secondary analysis of the ACCESS trial
Source: Sci Rep. 2022 Aug 25;12:14531. doi: 10.1038/s41598-022-18800-w (PMC9411156; doi:10.1038/s41598-022-18800-w)
Supplement: Supplementary file 2 — Supplementary Information 2. [file 41598_2022_18800_MOESM2_ESM.pdf]

Supplementary Method S1

Title: Implementation of a self-sampling HPV test for non-responders to cervical cancer screening in Japan: secondary analysis of the ACCESS trial

Authors: Misuzu Fujita[1,2], Kengo Nagashima[3,4], Minobu Shimazu[5], Misae Suzuki[6], Ichiro Tauchi[6], Miwa Sakuma[6], Setsuko Yamamoto[6], Hideki Hanaoka[5], Makio Shozu[7], Nobuhide Tsuruoka[8], Tokuzo Kasai[1], Akira Hata[1,9]

Affiliations: [1]Department of Health Research, Chiba Foundation for Health Promotion and Disease Prevention, Chiba 261-0002, Japan; [2]Department of Public Health, Chiba University Graduate School of Medicine, Chiba 260-8670, Japan; [3]Biostatistics Unit, Clinical and Translational Research Center, Keio University Hospital, Shinju-ku, Tokyo 160-8582, Japan; [4]Research Center for Medical and Health Data Science, The Institute of Statistical Mathematics, Tachikawa, Tokyo 190-8562, Japan; [5]Clinical Research Center, Chiba University Hospital, Chiba 260-8677, Japan; [6]Municipal Health Center, Department of Health and Welfare, Ichihara City, Ichihara, Chiba 290-0050, Japan; [7]Departments of Reproductive Medicine, Chiba University Graduate School of Medicine, Chiba 260-0856, Japan; [8]Yushudai Clinic, Ichihara, Chiba 299-0125, Japan; [9]Center for Preventive Medical Sciences, Chiba University, Chiba 260-0826, Japan

Endpoints of this secondary analysis

Proportion of self-sampling human papillomavirus (HPV) tests ordered
The percentage of the participants who ordered the self-sampling HPV test was calculated. The denominator is the intention to screen (ITS) in the self-sampling arm, which includes all participants who were assigned to the self-sampling arm. The numerator is the number of participants who ordered the test.

Proportion of self-sampling HPV tests ordered through the website
The percentage of the participants who ordered the test through the website, in which the denominator is all participants who ordered the test, and the numerator is those who ordered through the website, was calculated.

Proportion of self-sampling HPV test kits returned
The proportions of the participants who underwent an HPV test with two different denominators and one numerator were calculated. The denominators are ITS in the self-sampling arm and the number of participants who ordered self-sampling HPV tests. The numerator is the number of participants who return both a sample and a filled consent form.

Proportion of positive HPV tests
The percentage of participants with a positive HPV test result was defined as the detection of DNA of the 14 high-risk HPV types (types 16, 18, 31, 33, 35, 39, 45, 51, 52, 56, 58, 59, 66, and 68). The denominator is the number of participants who returned both a sample and a filled consent form. The numerator is the number of participants with a positive HPV test result.

Proportion of invalid HPV tests
The percentage of participants whose HPV test result was invalid was defined as the failure to detect DNA of the 14 high-risk HPV types and β-globin (internal cellular control). The denominator is the number of participants who returned both a sample and a filled consent form. The numerator is the number of participants whose HPV test result was invalid.

Time-to-event

Time to order the test
The period between the time that the second invitation letter was sent to the participants and the time that the self-sampling HPV test was ordered by the participants was calculated. The participants to be analyzed were those who ordered the test.

Time to send a kit
The period between the time that the self-sampling HPV test was ordered and the time that the kit was sent to the participants was calculated. The participants to be analyzed were those who ordered the self-sampling HPV test.

Time to return a sample since sending the kit
The period between the time that the kit was sent to the participants and the time a sample was returned was calculated. The participants to be analyzed were those who returned both a sample and a filled consent form, and these participants were named "participants A."

Time to return a sample since collecting it
The period between the time that a sample was collected by the participants and the time that

the sample was returned was calculated. The participants to be analyzed were those who returned both a sample and a filled consent form and reported the sample collection date.

Time to order the test to the laboratory
The period between the time that a sample was returned by the participants and the HPV test was ordered to the laboratory was calculated. The participants to be analyzed were "participants A."

Time for the laboratory to report the results
The period between the time that the HPV test was ordered to the laboratory and the results were reported by the laboratory was calculated. The participants to be analyzed were "participants A."

Time to send the results to the participants
The period between the time that the results were reported by the laboratory and the results were sent to the participants was calculated. The participants to be analyzed were "participants A."

Total time
The total period between the time that the second invitation letter was sent to the participants and the time that the HPV test results were sent to the participants was calculated. The participants to be analyzed were "participants A."

Incidence of adverse events

If adverse events were claimed by the participants, the percentage of those with adverse event(s) was calculated. The denominator is the number of participants who returned both a sample and a filled consent form. The numerator is the number of participants who reported each type of adverse event.
